# Exemplar Variance Supports Robust Learning of Facial Identity

**DOI:** 10.1037/xhp0000049

**Published:** 2015-04-13

**Authors:** Jennifer Murphy, Alberta Ipser, Sebastian B. Gaigg, Richard Cook

**Affiliations:** 1 Department of Psychology, City University London

**Keywords:** face learning, face recognition, internal feature advantage, exemplar variation, averaging

## Abstract

Differences in the visual processing of familiar and unfamiliar faces have prompted considerable interest in face learning, the process by which unfamiliar faces become familiar. Previous work indicates that face learning is determined in part by exposure duration; unsurprisingly, viewing faces for longer affords superior performance on subsequent recognition tests. However, there has been further speculation that exemplar variation, experience of different exemplars of the same facial identity, contributes to face learning independently of viewing time. Several leading accounts of face learning, including the averaging and pictorial coding models, predict an exemplar variation advantage. Nevertheless, the exemplar variation hypothesis currently lacks empirical support. The present study therefore sought to test this prediction by comparing the effects of unique exemplar face learning—a condition rich in exemplar variation—and repeated exemplar face learning—a condition that equates viewing time, but constrains exemplar variation. Crucially, observers who received unique exemplar learning displayed better recognition of novel exemplars of the learned identities at test, than observers in the repeated exemplar condition. These results have important theoretical and substantive implications for models of face learning and for approaches to face training in applied contexts.

Familiar and unfamiliar faces engage different types of visual processing (Burton & Jenkins, 2011; Hancock, Bruce, & Burton, 2000; Jenkins & Burton, 2011; Megreya & Burton, 2006). As faces become more familiar, observers are better able to match targets using their internal features (eyes, nose, mouth), the so-called “internal feature advantage.” In contrast, unfamiliar face matching is frequently based on external features, such as hairstyle and face shape (Ellis, Shepherd, & Davies, 1979; Osborne & Stevenage, 2008; Young, Hay, McWeeny, Flude, & Ellis, 1985). Familiar faces may also place lower demands on visual working memory (Jackson & Raymond, 2008) and are easier to detect under conditions of reduced attention (Jackson & Raymond, 2006), compared with unfamiliar faces. However, perhaps the most striking difference between familiar and unfamiliar face perception is the ease with which we can recognize new exemplars. In contrast with the effortless recognition of celebrities, colleagues, and friends, matching the faces of strangers across different photographic images can be remarkably difficult (Bruce et al., 1999; White, Kemp, Jenkins, Matheson, & Burton, 2014). For example, when asked to sort photographs of 2 individuals according to the identity of those depicted, observers perform poorly, frequently attributing the photographs to 8 or more different individuals (Jenkins, White, Van Montfort, & Burton, 2011).

Differences in the visual processing of familiar and unfamiliar faces have prompted considerable interest in face learning, the process by which unfamiliar faces become familiar. Previous evidence suggests that face learning is determined, at least in part, by the time observers spend viewing faces. Unsurprisingly, participants allowed to observe faces for 45 s each outperform those who view the same faces for 15 s (Memon, Hope, & Bull, 2003). Similarly, simple repetition of single facial images can improve subsequent recognition of actors in dynamic video stimuli (Roark, O’Toole, Abdi, & Barrett, 2006) and increase the strength of identity adaptation (Jiang, Blanz, & O’Toole, 2007), thought to be causally related to recognition ability (e.g., Dennett, McKone, Edwards, & Susilo, 2012). Crucially however, there has been further speculation that exemplar variation—experience of different exemplars of the same facial identity, such as that provided by a series of photographs—contributes to face learning, independently of viewing time.

Several leading accounts of face learning predict an exemplar variation advantage. For example, according to the pictorial coding model, familiar faces are recognized through comparison with previously stored instances of that face (e.g., Longmore, Liu, & Young, 2008). Having encountered multiple exemplars of a given face, observers are able to densely sample the potential instance space. Thereafter, the likelihood of a close match between a novel target exemplar and a previously stored instance is high, yielding superior face recognition performance. In contrast, an observer who has previously encountered only a few exemplars of a facial identity must rely on a sparse sampling of the instance space, and may therefore struggle to match the target to a previously stored instance.

A second closely related model is the averaging hypothesis (Benson & Perrett, 1993; Burton, Jenkins, Hancock, & White, 2005; Jenkins & Burton, 2011). According to this view, exemplar variation allows the visual system to form a robust average of that facial identity. Transient differences including variation in lighting, shadow, hairstyle, and expression are discounted, leaving a stable representation of permanent, reliable features. Crucially, (a) accurate recognition of the target face upon subsequent encounters is thought to be directly related to the quality of the average formed (e.g., Jenkins & Burton, 2008), and (b) average estimates derived from many observations are likely to better approximate the true parameter value than estimates derived from only a handful of observations of comparable quality.

Despite its theoretical significance, however, the exemplar variation hypothesis currently lacks empirical support. Specifically, there is little evidence that exemplar rich experience supports better face learning, when viewing time is equated. In the absence of empirical evidence in favor of the exemplar variation hypothesis, it is possible that the strength of face learning is determined solely by viewing time.^1^ The present study, therefore, adopted a two-stage training-test procedure to determine whether exposure to many unique exemplars of a target face (“unique exemplar learning”) supports superior recognition, compared with equivalent exposure to a limited number of exemplars (“repeated exemplar learning”). Similar recognition performance following both types of training would indicate that face learning is determined not by exemplar variation, but by the time spent viewing the trained identities, challenging a key assumption of the pictorial coding and averaging accounts.

## Method

### Participants

Fifty healthy adults (19 males, *M*_age_ = 29.5 years, *SD*_age_ = 8.1 years) participated in this experiment in return for a small honorarium. Participants were randomly allocated to the different training conditions in equal numbers. Four participants, 2 in each condition, were replacements. Two of the replaced observers scored at chance level at test, 1 reported prior familiarity with a learned identity, and 1 was an outlier in terms of response latencies. All participants had normal or corrected-to-normal vision, gave informed consent, and were fully debriefed upon task completion. Sample size was determined a priori using power analysis assuming a large effect size (Cohen, 1988). Ethical clearance was granted by the local ethics committee, and the study was conducted in accordance with the ethical standards laid down in the 1964 Declaration of Helsinki.

### Training

**Figure 1 fig1:**
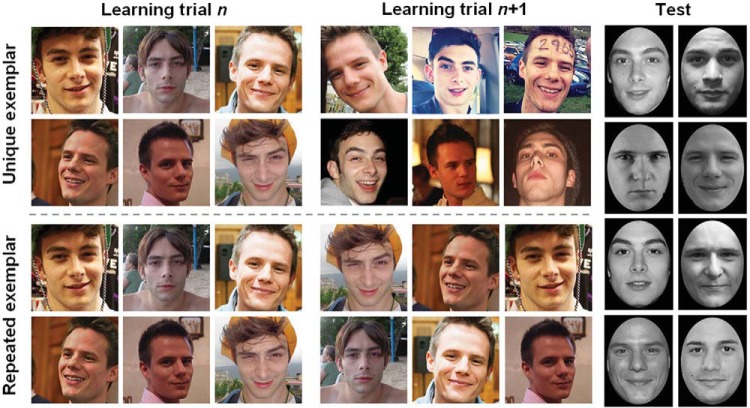
Examples of the training images and illustration of the learning manipulation (left). Observers in the unique exemplar learning condition were exposed to 6 new photos of each learned identity on each training trial. In the repeated exemplar condition, observers were exposed to the same 6 photos on every learning trial. Examples of the test images (right). The individuals whose faces appear here have consented to be included in the authors’ database from which these images are taken.

Participants completed one of two different face learning procedures, each comprising 16 trials. Each training trial presented 48 facial images simultaneously in a 6 × 8 array. Training arrays were presented for 48 s, during which observers were free to inspect the images as they wished. Following array offset, a prompt appeared to judge the number of identities represented within the array, as accurately as possible. Unbeknown to observers, the 48 images were in fact always taken from only 8 individuals, training arrays comprising 6 images of each. The same 8 individuals—the to-be-learned identities—were shown on every training trial. The training faces sampled a broad range of poses, expressions, hairstyles, lighting conditions, and camera parameters (see Figure 1).[Fig-anchor fig1]

Observers in the unique exemplar condition saw a novel set of 48 images on each training trial. Across the procedure, they were therefore exposed to 96 photos of each of the 8 to-be-learned identities, 6 novel exemplars of each identity on each trial. Observers in this condition viewed different combinations of exemplars, chosen at random by the program, on each of the 16 training trials. Observers in the repeated exemplar condition, however, were exposed to the same 48 images on all 16 training trials. Consequently, observers saw only 6 photos of each of the 8 to-be-learned identities. Each observer in this condition was trained on a different set of exemplars, chosen at random from the 96 images comprising each of the 8 identity sets. That a novel combination of 48 images was selected for each participant guarded against the possibility of systematic bias. While the same 48 images were repeated, the position of the exemplars in the 6 × 8 array was randomized across the 16 learning trials. In all other respects, the two learning conditions were identical.

Training images were cropped to square aspect ratios subtending 3° vertically when viewed in the array at a distance of 60 cm. The to-be-learned identity was the only face visible in each image and was central and prominent. In all other respects, the raw photographic images were left unaltered. Images showing the to-be-learned identities wearing glasses or sunglasses were intentionally avoided. The next trial commenced only once a response had been recorded. Four practice trials incorporating cartoon faces from *The Simpsons* preceded the learning procedure. All experimental programs were written in MATLAB using Psychtoolbox (Brainard, 1997; Pelli, 1997), and presented on a Dell 17-in. Liquid-crystal display (LCD) monitor at 60-Hz refresh rate.

### Test

Immediately after training, all participants completed the same test procedure to assess their recognition of the 8 learned identities. Unlike the training faces, test faces were presented in greyscale and were cropped to exclude external features, thereby removing the cues typically used to recognize unfamiliar faces (Ellis et al., 1979; Osborne & Stevenage, 2008; Young et al., 1985). Trials briefly (for 1,000 ms) presented a single face centrally, followed by a prompt to judge whether the identity was or was not encountered during the learning phase (old or new). Half of the facial images presented during the test depicted the 8 learned identities; however, none of the exemplars were used during the training phase. The remaining test images were exemplars of 8 novel identities not encountered previously. Test faces were presented in greyscale and subtended 6.5° vertically when viewed at 60 cm. All test faces were shown in frontal view and had approximately neutral expressions. The old and new test images were closely matched, both in terms of the identities depicted and their presentation. In total, the test procedure comprised 160 trials: 5 novel exemplars of the 8 trained identities and 5 exemplars of 8 novel identities were presented twice each. The test was preceded by 8 practice trials incorporating cartoon faces from *The Simpsons.*

## Results

**Figure 2 fig2:**
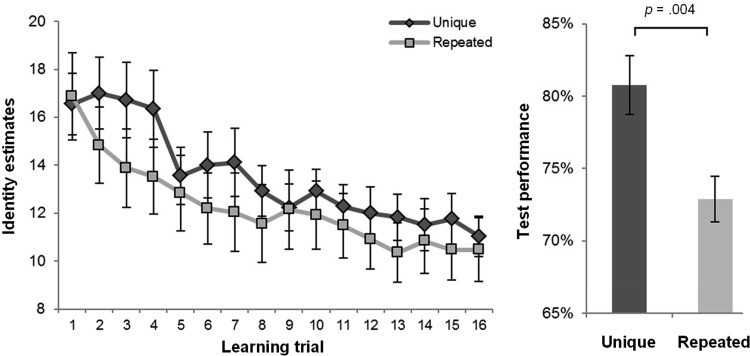
Results from the training phase (left). Observers in both conditions overestimated the number of individuals present in the training arrays, particularly at the start of the procedure. Identity estimates declined steadily thereafter indicative of learning. Results from the test phase (right). Participants were briefly presented cropped greyscale facial images, and asked whether or not the identity depicted was encountered during the learning phase (“old” or “new”). In accordance with the exemplar variation hypothesis, unique exemplar learning supported better recognition than repeated exemplar learning. Error bars indicate ±1 standard error of the mean.

The identity estimates from the training phase (see Figure 2) were analyzed using analyses of variance (ANOVA) with trial (1:16) as a within-subjects factor and learning condition (unique exemplar, repeated exemplar) as a between-subjects factor. The analysis revealed highly significant linear, *F*(1, 48) = 37.250, *p* < .001, η^2^ = .437, and quadratic trends, *F*(1, 48) = 15.065, *p* < .001, η^2^ = .239, indicative of learning across trials. Neither the linear, *F*(1, 48) = .249, *p* = .620, η^2^ = .005, nor quadratic, *F*(1, 48) = .224, *p* = .638, η^2^ = .005, trends varied as a function of learning condition, indicating that the change in identity estimates across the training procedure was broadly comparable for the two groups. The main effect of learning condition was not significant, *F*(1, 48) = .558, *p* = .459, η^2^ = .011, suggestive of similar identity estimates overall. The mean identity estimates did not differ between the learning conditions on the first, *t*(48) = .144, *p* = .886, or last trials, *t*(48) = .355, *p* = .724. Despite the learning observed, the identity estimates of both the unique exemplar, *t*(24) = 16.249, *p* < .001, and repeated exemplar, *t*(24) = 13.226, *p* < .001, groups still exceeded the true number (8 identities) on the final training trial.[Fig-anchor fig2]

The crucial differences between the groups were seen at test. As predicted by the exemplar variation hypothesis, the observers who received unique exemplar learning (*M* = 80.8%, *SD* = 10.2%) outperformed those who received repeated exemplar learning (*M* = 72.9%, *SD* = 8.0%), *t*(48) = 3.044, *p* = .004. This difference corresponds to a Cohen’s *d* of 0.80, indicative of a large effect (Cohen, 1988). The advantage of unique exemplar learning (*M* = 77.9%, *SD* = 14.7%) over repeated exemplar learning (*M* = 66.0%, *SD* = 13.6%), was only present for hits; that is, correct old responses in the presence of an old stimulus, *t*(48) = 2.993, *p* = .004. No advantage of unique exemplar learning (*M* = 83.7%, *SD* = 11.1%) over repeated exemplar learning (*M* = 79.9%, *SD* = 18.0%) was observed for correct rejections; that is, new responses in the presence of a new stimulus, *t*(39.841) = .908, *p* = .369 (corrected for inequality of variance). Of note, simple correlation analyses revealed that the identity estimates from the final training trial, an index of the performance achieved by each observer on completion of the training task, correlated significantly with test performance for the unique exemplar condition, *r* = −.503, *p* = .010, but not for the repeated exemplar condition, *r* = .013, *p* = .952.

## Discussion

The present study compared the effects of unique exemplar face learning—a condition rich in exemplar variation—and repeated exemplar face learning—a condition that equates viewing time, but constrains exemplar variation. During training, observers in both learning conditions overestimated the number of individuals present, particularly at the start of the procedure, when the identities were novel. As the faces became more familiar, both groups found it easier to recognize the commonalities across different exemplars, and their identity estimates became progressively more accurate. Crucially, however, observers who received unique exemplar learning displayed better recognition of novel exemplars of the learned identities at test, than observers in the repeated exemplar condition. These results have important theoretical and substantive implications for models of face learning and for approaches to face training in applied contexts. Crucially, these results confirm for the first time that face learning is determined not only by the time spent viewing a face, but also the degree of exemplar variation experienced by the learner.

The finding that observers overestimate the number of identities present in arrays comprising unfamiliar faces is consistent with previous reports (Jenkins et al., 2011), and further underscores the challenges posed by unfamiliar face matching (Bruce et al., 1999; White et al., 2014). However, in both learning conditions, identity estimates declined sharply across the first 8–10 training trials, revealing that a degree of familiarity can be acquired rapidly based on relatively little exemplar variation. Interestingly, the identity estimates from the final training trial, an index of the performance achieved by each observer on completion of the training task, correlated significantly with test performance for the unique exemplar condition, but not for the repeated exemplar condition. This unexpected finding raises the possibility that the nature of the leaning observed in the two conditions was qualitatively different. In light of previous findings (e.g., Osborne & Stevenage, 2008), we speculate that observers in the repeated exemplar condition were disproportionately reliant on the external facial features during the training procedure, whereas those allocated to the unique exemplar condition grew more adept at distinguishing faces from their internal features. When forced to discriminate faces based solely on internal features at test, performance therefore correlated with individual differences in unique exemplar learning, but not with the variability seen in repeated exemplar learning.

The present results have interesting implications for the study of moving faces. When considered as a time-series of different facial poses and expressions, it is clear that even a short sequence of facial motion contains many unique exemplars. Viewing moving faces might therefore be expected to convey a face learning advantage relative to the observation of a few static images (O’Toole, Roark, & Abdi, 2002). Paradoxically however, many studies have found little or no evidence for such a motion advantage when learning new faces (e.g., Bonner, Burton, & Bruce, 2003). One possibility is that the rigid and nonrigid movements present within naturalistic sequences of facial motion may be relatively constrained, and therefore fail to yield sufficient exemplar variation to generate a clear learning advantage.

Overall, the present findings show for the first time that face learning is determined not only by the time spent viewing a face, but also the degree of exemplar variation experienced by the learner. That unique exemplar learning affords recognition performance superior to repeated exemplar learning, confirms that exemplar variation contributes to face learning in human vision. These results suggest that face training applications should seek to maximize exemplar variation to optimize face learning.

## Footnotes

1 For example, having experienced how familiar faces appear in different poses and lighting situations during the course of development, adults may be able to extrapolate from, or interpolate between, a handful of exemplars of a novel target face. Should observers be able to generate variation endogenously, through the application of previously learned image transformations, the information conveyed by additional unique exemplars may be redundant.
